# Inhomogeneities in Network Structure and Excitability Govern Initiation and Propagation of Spontaneous Burst Activity

**DOI:** 10.3389/fnins.2019.00543

**Published:** 2019-05-31

**Authors:** Samora Okujeni, Ulrich Egert

**Affiliations:** ^1^Biomicrotechnology, IMTEK – Department of Microsystems Engineering, University of Freiburg, Freiburg, Germany; ^2^Bernstein Center Freiburg, University of Freiburg, Freiburg, Germany

**Keywords:** burst initiation zones (BIZ), neuronal network, inhomogeneity, synchronous bursting event, network structure, microelectrode array (MEA), mesoscale network architecture

## Abstract

The mesoscale architecture of neuronal networks strongly influences the initiation of spontaneous activity and its pathways of propagation. Spontaneous activity has been studied extensively in networks of cultured cortical neurons that generate complex yet reproducible patterns of synchronous bursting events that resemble the activity dynamics in developing neuronal networks *in vivo*. Synchronous bursts are mostly thought to be triggered at burst initiation sites due to build-up of noise or by highly active neurons, or to reflect reverberating activity that circulates within larger networks, although neither of these has been observed directly. Inferring such collective dynamics in neuronal populations from electrophysiological recordings crucially depends on the spatial resolution and sampling ratio relative to the size of the networks assessed. Using large-scale microelectrode arrays with 1024 electrodes at 0.3 mm pitch that covered the full extent of *in vitro* networks on about 1 cm^2^, we investigated where bursts of spontaneous activity arise and how their propagation patterns relate to the regions of origin, the network’s structure, and to the overall distribution of activity. A set of alternating burst initiation zones (BIZ) dominated the initiation of distinct bursting events and triggered specific propagation patterns. Moreover, BIZs were typically located in areas with moderate activity levels, i.e., at transitions between hot and cold spots. The activity-dependent alternation between these zones suggests that the local networks forming the dominating BIZ enter a transient depressed state after several cycles (similar to Eytan et al., 2003), allowing other BIZs to take over temporarily. We propose that inhomogeneities in the network structure define such BIZs and that the depletion of local synaptic resources limit repetitive burst initiation.

## Introduction

The patterns of activity in neuronal networks depend on numerous factors, such as the networks size, density, clustering, axonal bundling, cell type composition, plasticity, etc. Previous studies have sought to address some of these aspects and their interaction with neuronal networks grown on microelectrode arrays (MEA) with up to several thousand electrodes (Obien et al., 2014), but mostly with passive planar arrays of 60-256 electrodes (for review see Stett et al., 2003; Liu et al., 2012). Studies using such MEAs sample a network’s activity through the limited spatial window and resolution of the array, generally more or less covering the network’s center, yet tend to interpret the findings to be representative for the network as a whole. This undersampling, however, could lead to considerable misinterpretation if critical structures of connectivity and activity dynamics were located in the outskirts of a network. There, activity-dependent homeostatic regulation of neurite growth might lead to special connectivity motifs because of anisotropic connection opportunities for neurons at the boundary. This would lead to higher abundance of recurrent connectivity motifs between peripheral neurons, with potentially profound impact on activity.

How spontaneous activity arises in cultured networks is not fully understood. On most electrodes, synchronous bursting events (SBE) seem to arise suddenly from very sparse background activity, while only few electrodes record tonically active neurons. It was proposed that SBE initiation reflects a threshold processes after gradual activity buildup in a network (Maeda et al., 1995; Giugliano et al., 2004; Eytan and Marom, 2006). Network models, in turn, attribute an important role to pacemaker neurons in governing the observed SBE dynamics (Gritsun et al., 2010). In addition, using special MEA designs with electrodes at the boundary and in the center of large-scale networks of 450,000 neurons on 380 mm^2^, it was shown that if networks are large enough they may enter in a state of continuously circulating SBE activity (Keren and Marom, 2016) that is only seemingly split into distinct events because the small observation window of small, central MEAs, would be traversed by activity only occasionally. Indeed, looking at propagation patterns recorded with smaller MEAs suggests that SBEs mostly seem to originate outside of the array area (Volman et al., 2005; Okujeni et al., 2017; Pasquale et al., 2017). Investigations of abstract networks further suggest considerable influence of peripheral network nodes (Zhang et al., 2016).

The relative contribution of inhomogeneity resulting from compensatory growth at the boundary may, however, vary with the overall homogeneity of the network, i.e., in networks with homogeneous distributions of neurons and neurites boundary effects may have higher impact than in networks with an overall more irregular structure. We have previously shown that the inhomogeneity of a network drastically influences the patterns of activity it generates (Okujeni et al., 2017).

To analyze the role of the network’s boundary and its central connectivity for the initiation and subsequent recruitment of the network during SBEs, we used very large MEAs with 1024 electrodes (1k-MEA) covering whole networks. We investigated how the network boundary and inhomogeneities in network structure and excitability relate to the initiation and propagation of SBEs. We examined how contributions of the boundary area depend on the overall structure of the network by modifying proteine kinase C (PKC) activity, which changes neurite extent and cell migration, leading to more homogeneous or more inhomogeneous network architectures.

We show that SBEs in networks of about 150,000 neurons arise from distinct burst initiation zones (BIZ) and are followed by an overall depression of network activity. BIZs were most frequently located close to the network boundary in control networks and almost exclusively so in network with more homogeneous growth and connectivity. BIZs were not congruent with regions generating high firing rates during bursts but were located in areas of intermediate activity. Our findings show that adequately addressing the boundaries and inhomogeneous structures in neuronal networks is essential to interpret the patterns of SBE activity correctly, and thus to understand how structural and functional network properties relate to each other.

## Materials and Methods

### Cell Culture Techniques

Primary cortical cell cultures were prepared on different MEAs (Multi Channel Systems, Germany (MCS); electrode grid layout/pitch distance (μm): 8 × 8/200, 6 × 10/500, 16 × 16/200, 32 × 32/300). MEAs were coated with polyethylene-imine (150 μl 0.2% aqueous solution; Sigma-Aldrich, Germany) for cell adhesion. Cell cultures were prepared following Shahaf and Marom (2001). Cortical tissue was prepared from brains of neonatal Wistar rat pups of either sex, minced with a scalpel and transferred into phosphate buffered saline (Invitrogen, Germany). Tissue pieces were incubated with trypsin (isozyme mixture, 0.05%, 15 min at 37°C; Invitrogen) and proteolysis was subsequently stopped with horse serum (20%; Invitrogen). DNase (type IV, 50 μg/ml; Sigma-Aldrich) was added to eliminate cell trapping in DNA strings if needed. Cells were dissociated by trituration with a serological pipette, centrifuged (5 min, 617 *g*) and resuspended in growth medium (Minimal Essential Medium supplemented with 5% heat-inactivated horse serum, 0.5–1 mM L-glutamine, 20 mM glucose and 20 μg/ml gentamycin (all from Invitrogen); 1 ml/pup). Cells were counted with an automated cell counter (CASY, Schärfe Systems GmbH, Germany) and seeded at ∼300.000 cells per culture, resulting in a density of ∼1500 neurons/mm^2^ at 1 days *in vitro* (DIV). Networks developed in 1 ml growth medium in a humidified incubator (5% CO_2_. 37°C). Animal handling and tissue preparation were done in accordance with the guidelines for animal research at the University of Freiburg.

### PKC Modulation

To assess the influence of mesoscale network architecture on the initiation and propagation of SBEs, we inhibited PKC activity during development as described previously (Okujeni et al., 2017). This lead to a more homogeneous arrangement of neurons and neurites in the network. PKC inhibitor Gödecke6976 (Gö6976, 1 μM, Sigma-Aldrich) was dissolved in dimethyl sulfoxide (DMSO, Sigma-Aldrich) and added to the culture medium directly after cell preparation. The maximal concentration of DMSO in the growth medium was 0.1%.

### Immunohistochemical Stainings

Neuronal morphology on standard MEAs was examined by immunocytochemical staining of microtubule-associated protein 2 (MAP2) (Chicken-anti-MAP2; 1:500; Abcam, United States) expressed in dendrites and somata. Large-scale images spanning the full network (120 × 120 mm) were taken at 10-fold resolution (0.645 pixel/μm; Examiner Z1 microscope, Zen software 2015, Carl Zeiss, Jena, Germany) and processed by background subtraction (750 × 750 pixel median filtering using Zen).

### Extracellular Recording and Analyses

Multi-unit spike activity was recorded from MEAs (MEA1060-BC, USB-MEA256-System and USB-MEA30-1024-System amplifiers; Multi Channel Systems, 25 kHz sampling frequency, 12 bit) under culture conditions (37°C, 5% CO_2_) and acquired with MCRack software (Multi Channel Systems; versions 3.3 – 4.5). Recordings of individual networks lasted at least 1 h. Action potentials (AP) were detected with a threshold set to -5 standard deviations (STD) of the high-pass filtered baseline signal (Butterworth 2nd order high pass filter, 200 Hz cut-off; detection dead time 2 ms).

Raw data from MEA recordings was imported into Matlab using MEA-Tools (Egert et al., 2002) and the FIND toolbox (Meier et al., 2008). Spontaneous SBEs were detected as follows: Series of spikes with consecutive inter-spike intervals smaller than a threshold value (100 ms) were detected as bursts. SBEs were defined as periods in which a predefined fraction of electrodes showed simultaneous bursts (>5% of coactive sites). To account for buildup and fading phases of SBEs, spikes within a time windows of 50 ms prior to and following this SBE core were included into the SBE. SBE strength was calculated as the average number of spikes detected per electrode between SBE onset and offset.

Coactivity over time was calculated as the fraction of coactive sites within a sliding time window of 1, 10, or 100 ms. SBE-onset-triggered average coactivity was calculated excluding spikes of the last and next SBE, with an additional safety window of 500 ms after, respectively before the offset and onset of these SBEs, to account for fading and buildup phases not related to the current event.

We determined the ratio between average firing rates (AFR) at onset electrodes and remaining electrodes with respect to SBE onset to investigate the activity dynamics associated with SBE onset. We calculated this ratio as the AFR for the earliest 10 electrodes in each SBE divided by the AFR of the remaining electrodes using a window of ± 10 s around SBE onset as described above (bin width 10 ms). For this calculation we also excluded spikes of the last and next SBE, with an additional safety window of 500 ms after, respectively before the offset and onset of these SBEs, to account for fading and buildup phases not related to the current event.

The initiation and propagation of activity was characterized by the rank order in which respective first spikes were detected at each electrode (first spike rank order, FSRO). To reduce noise-related jitter, spatial rank order maps were smoothed by 3 × 3 median filtering. The first ten ranks in the resulting map were defined as the *SBE onset electrodes* and the arithmetic means of their x and y coordinates was used to define the onset location. To identify BIZs that repeatedly triggered SBEs, we determined clusters in the spatial distribution of onset locations derived from many SBEs by centroid clustering with a cutoff of 1 mm. *BIZ electrodes* were defined as the ten most frequently present electrodes within the onset electrodes of each cluster. The BIZ position was defined as the arithmetic means of the x and y coordinates of these BIZ electrodes.

Relative activity levels at electrodes were calculated as the AFR over the recording period normalized by the mean AFR of all electrodes with spike activity. We also compared activity levels in BIZs relative to the highest 25% of AFRs on the MEA. Maps showing relative activity levels were smoothed by 3 × 3 median filtering. Values in the text represent mean and standard error of mean (SEM) across electrodes. Significance was assessed by independent Student’s *t*-test.

To determine whether BIZs were more active when initiating SBEs, we compared the burst strength at BIZ electrodes (number of burst spikes per electrode) when BIZs were actively initiating or passively recruited during SBEs. To compare the activity across BIZs in active and passive roles we calculated burst strength distributions for the nine most prominent BIZs in each network. For each of these BIZs, we compared their relative burst strength for the active condition against the situation when any of the other BIZ triggered a burst. For an overall comparison, we z-scored burst strengths for each BIZ individually and then tested for differences between the distribution of pooled z-scored burst strengths from all BIZs in the active condition against the corresponding distribution in the passive condition. Significance of relative changes was tested with independent Student’s *t*-test.

Similarity of SBE propagation patterns was calculated by correlation of FSRO patterns (without smoothing). Correlation values for SBEs triggered by the same BIZ are presented in the text as mean and STD over all eligible SBE pairs in the entire correlation matrix. Significance was assessed by paired Student’s *t*-test.

Recordings with 8 × 8, 6 × 10 and 16 × 16 MEAs were made from at least 10 networks per PKC condition. Data from 1k-MEAs include two recordings at different developmental stages, from two networks per PKC condition.

## Results

In the past decades, MEAs have been extensively used to assess the activity dynamics of neuronal networks in cell culture as they allow to record from many neurons as a representative sample. At 1 DIV, we typically determined ∼150,000 neurons that adhered onto a surface area of about 1 cm^2^ and started to form a network. Given the limited spatial extent of most commercially available MEAs (typically a few mm), it is obvious that for such networks only a local region, in most cases the center, of the network is actually sampled. Using 1k-MEAs we assessed the dynamics in critical regions like the boundary and analyzed their role for SBE initiation and the development of propagation patterns.

### Incomplete Sampling of SBE Initiation and Propagation With Standard MEAs

**FIGURE 1:**
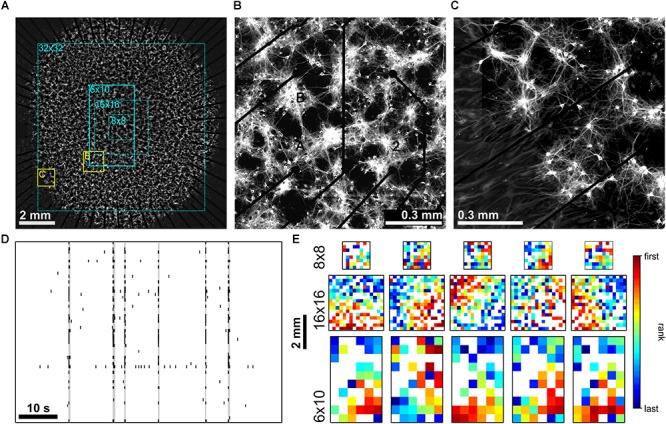
**(A)** PKC^N^ network (∼150,000 neurons on ∼100 mm^2^) on a 6 × 10 MEA (500 μm pitch). Neurons were stained for MAP2 expressed in dendrites and somata (22 DIV). These networks formed local clusters and sparser regions in between clusters. The electrode area (solid cyan rectangle) covered only the central part of the network. Dotted cyan rectangles outline the spatial extent that 8 × 8 (200 μm pitch), 16 × 16 (200 μm pitch) and large-scale 32 × 32 (300 μm pitch) MEAs would cover. **(B)** Zoom-in on the network region recorded by the MEA (yellow rectangle marked B in A). **(C)** Clustering was also present along the boundary of the network (yellow rectangle marked C in A). **(D)** Representative MEA Recordings of synchronous bursting events (SBE) activity at 22 DIV from the network in A. **(E)** Exemplary SBE propagation patterns from networks grown on 8 × 8 (top row, 0.2 mm electrode pitch), 16 × 16 (middle row, 0.2 mm pitch) and 6 × 10 (bottom row, network in A, 0.5 mm pitch) MEAs recorded at 20–30 DIV. Color codes for the rank order of the first spike on each electrode in the course of SBEs. Estimation of SBE origins from the radius of the approximately circular wave front suggests that these were mostly located outside of the area recorded.

We compared spike activity in networks of cultured cortical neurons using MEA recordings with different electrode array layouts [commercially available arrays with 8 × 8 (1.2 × 1.2 mm^2^), 6 × 10 (2.5 × 4.5 mm^2^) and 16 × 16 (3.0 × 3.0 mm^2^) electrodes and a custom built array with a 32 × 32 electrodes (9.3 × 9.3 mm^2^)]. The latter covered almost the full area of cultured networks of approximately 1 cm^2^ (**Figure 1A**) and thus allowed simultaneous sampling of activity of network regions in the center (**Figure 1B**) and boundary (**Figures 1A,C**). Without modulation of PKC activity (PKC^N^), neurons formed moderately clustered networks with more or less regularly spaced local neuron clusters and sparser regions in between. As reported earlier (Okujeni et al., 2017), these networks generated SBEs (**Figure 1D**) that started in a localized network area and then recruited large parts of the network. Recruitment patterns derived by ranking electrodes according to timing order of first spikes recorded in the course of the SBE (FSRO) were used to characterize the propagation of activity across the network. The appearance of these patterns depended on the MEA layout. With small 8 × 8 electrode arrays (**Figure 1A**, dotted cyan rectangle), recruitment patterns did not show a clear wave front of activity propagation but rather indicated a more or less random recruitment of neurons (**Figure 1E**), as reported earlier (Shahaf et al., 2008). Recruitment patterns assessed with larger 6 × 10 electrode arrays (**Figure 1A**, solid cyan rectangle) indicated an approximately circular propagation front and thus that SBEs were actually initiated within a local BIZ. Given the percentage of network coverage provided by different arrays (8 × 8: 2%, 16 × 16: 9%, 6 × 10: 11%; 32 × 32: 87%; **Figure 1A**), we expected that only a corresponding fraction of SBEs would be initiated within the recording area if BIZ were randomly distributed throughout the network. For small arrays, earliest activity during SBEs was indeed often recorded from electrodes at the array boundary, suggesting many BIZs outside of the recording area (**Figure 1E**).

### Full Network Recordings With Large-Scale MEAs

**FIGURE 2:**
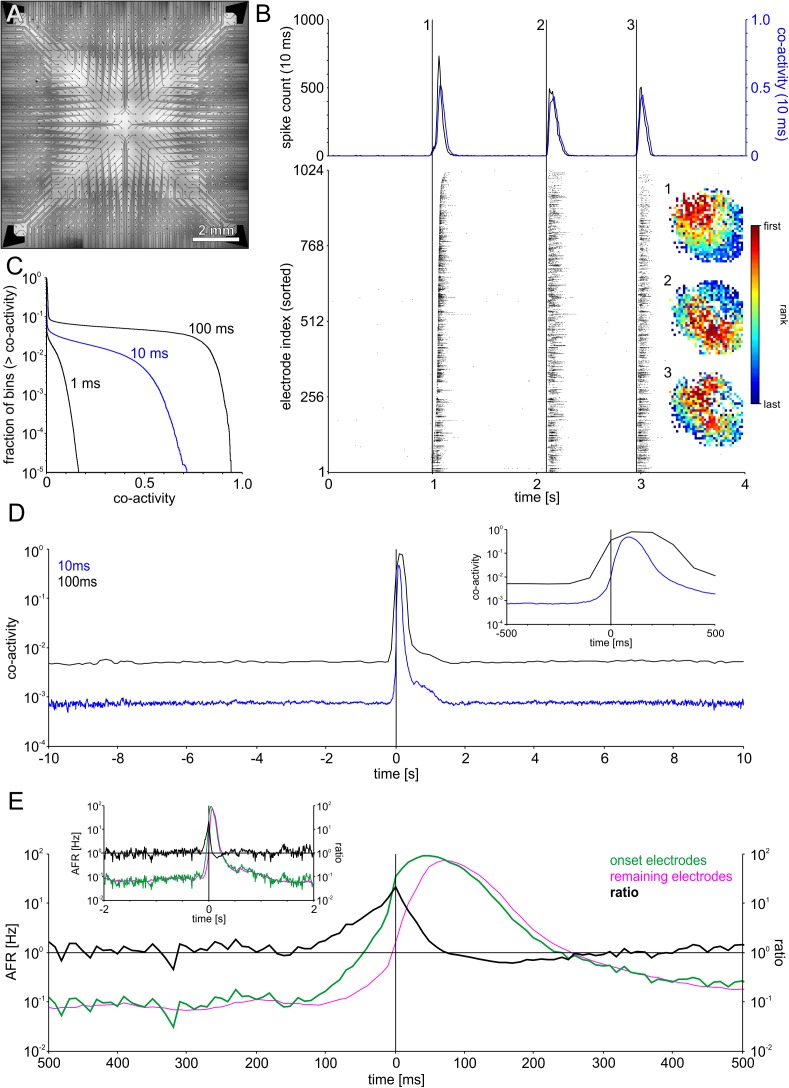
Recording with a large-scale electrode array that spanned the entire network area revealed SBEs starting in different regions **(A)** Layout of 1k-MEAs (1024 electrodes, 0.3 mm pitch). **(B)** Successive SBEs initiated at three different sites in an example network followed specific propagation patterns. The subsequent recruitment of the network led to a steep increase and subsequent decline of the global firing rate during SBEs (upper panel). Co-activity captured correlated firing within the network as the fraction of the network active within 10 ms sliding windows. Co-activity dropped to almost zero between peaks, indicating that SBEs are separate events, rather than modulations of sustained network-wide activity. The different propagation patterns are revealed by mapping the rank order of the first spike on each electrode during SBEs. **(C)** Cumulative frequency distributions indicating the fraction of bins whose co-activity was above a specific level, shown for different bin widths (1, 10, and 100 ms). Even for large bin widths, periods of network wide activity constitute only a small fraction, e.g., for 100 ms bins, co-activity was > 0.5 in ∼5% of the bins. **(D)** SBE onset-triggered co-activity (window size 10 ms) averaged across all SBEs. In-between SBEs, co-activity was < 0.01 (*N* = 1887 SBEs). The inset shows a smaller time window surrounding SBE onset. **(E)** SBE-onset-triggered AFR ratio between the varying set of onset electrodes (first ten electrodes) in SBEs and the respective remainder of the network. AFRs at onset electrodes exceeded the remaining network starting about 150 ms prior to SBE onset time and peaked at SBE onset. The subsequent decrease of AFRs at onset sites below the AFRs in the rest of the networks within a time window of about 200 ms indicates depressed activity in onset areas after SBE initiation. The inset shows a larger time window surrounding SBE onset.

From recordings with small MEAs placed in the center of a network it is not possible to determine whether SBEs arise as a modulation of reverberating background activity or reflect independent spontaneous activation processes. To gain a full picture of SBE generation and propagation we recorded networks with 32 × 32 electrode arrays (1k-MEA, **Figure 2A**) that spanned the entire network area (see **Figure 1**). With 1024 electrodes and estimated 150,000 neurons, a sampling ratio of about 1:150, we could assess potential sustained background dynamics in all areas of the network. SBEs in spontaneous network activity had temporal dynamics comparable to recordings from smaller MEAs with incomplete network coverage (**Figure 2B**). The full scale network recordings (4 networks) showed that these SBEs were not restricted to particular areas of the network, but typically recruited the whole network.

To assess collective background activity, we determined the level of coactivity between neurons in sliding time windows of 10 ms (**Figure 2B**, top). During SBEs, coactivity increased to high levels conjoint with the global spike rate. The level of coactivity crucially depends on the time window used to assess the fraction of coactive neurons in the network (**Figure 2C**) but the fraction of time in which the network was in a state of significant coactivity was below 10% even for time windows of 100 ms. In-between SBEs, coactivity levels were very low providing no indication for sustained propagating network activity (**Figure 2D**), suggesting that SBEs were delimited events. SBE onsets were preceded by an activity build-up phase of about 150 ms, during which AFRs increased at onset electrodes relative to the remaining electrodes (**Figure 2E**).

### Initiation and Propagation of Activity in Moderately Clustered Networks

**FIGURE 3:**
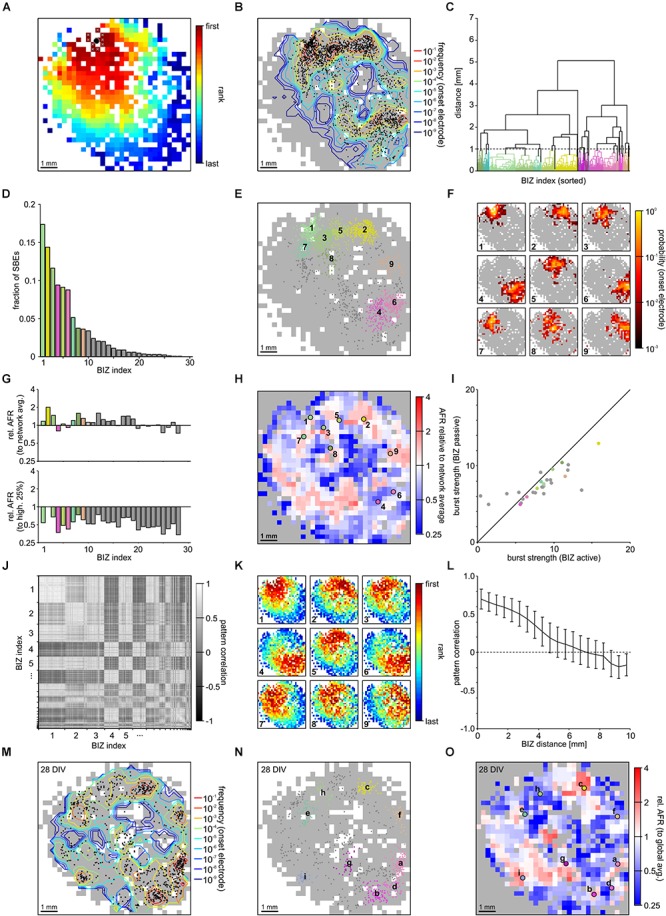
Synchronous bursting events dynamics in a PKC^N^ network at 21 DIV (NW_1_). **(A)** SBEs were mostly initiated in compact regions and propagated across the network from there (3 × 3 median filter smoothing). The black spot marks the means of the x and y coordinates of the first ten recruited electrodes that was defined as onset location. **(B)** Onset locations were distributed across large areas of the network but clustered in certain regions reflecting distinct BIZs (*N* = 1887 SBEs, *t* = 165 min; 11.4 SBE/min). Contour lines indicate the frequency with which individual electrodes were among onset electrodes (smoothed by 3 × 3 median filtering). **(C)** BIZs were identified by spatial centroid clustering of onset locations (cut-off at 1 mm distance between onset locations). **(D)** Burst initiation was dominated by only few BIZs. For clarity only the nine most frequent BIZ are color coded according to their position. **(E)** Mapping individual SBE onset locations shown in B to their respective BIZ (color code as in D) indicates that dominating BIZ lay close to but not at the network boundary. **(F)** BIZs reflected the centers of burst onset regions, which were mostly compact but not confined to extremely localized positions. The maps show the probability by which electrodes were among the first ten onset electrodes of bursts starting within the dominating BIZs. **(G)** Average relative activity levels at BIZ electrodes (ratio of the mean AFR at BIZ electrodes and of all other electrodes with spike activity). Activity levels were often slightly above network average in BIZs but always lower than the 25% of highest AFRs. **(H)** Map of relative activity levels (ratio between the AFR at individual electrodes and network AFR during SBEs). BIZs appeared mostly located on transitions between hot and cold spots. **(I)** Median burst strength at BIZ electrodes when driving SBEs (active) or recruited during SBEs initiated by other BIZs (passive). Activity in the major BIZs was only slightly higher when they initiated SBEs or were passively recruited into the SBE. **(J)** Similarity between propagation patterns was determined as the correlation of FSRO. Sorting correlation coefficients according to BIZ assignment reveals a high correlation between propagation patterns originating at the same BIZs. **(K)** Average propagation patterns elicited by the nine most frequent BIZs. **(L)** The correlation between propagation patterns decreased with increasing distance between BIZs, which yielded slightly anti-correlated patterns for BIZ located at opposite sides of the network. **(M)** The distribution of SBE onset locations for the same network recorded one week later was highly similar (28 DIV, *N* = 814 SBE, *t* = 61 min; 13.3 SBE/min). **(N)** The overall distribution of BIZs was mostly preserved during development (colors code as in D). Comparing letter sequence (reflecting decreasing frequency) to the numbers in E shows, however, that their influence had changed. **(O)** Relative activity levels across the MEA displayed comparable patterns at 21 and 28 DIV.

We used FSRO maps derived from recordings of two moderately clustered networks grown on 1k-MEAs to capture the full picture of SBE initiation and propagation during spontaneous activity dynamics. SBEs originated within a localized network region and then spread across the entire network (network 1 (NW_1_): **Figure 3A** and network 2 (NW_2_): Supplementary Figure S1). We defined the first ten ranks in recruitment order (after spatial 3 × 3 median filtering) as *onset electrodes* of an individual SBE (**Figure 3A**, white crosses in the upper left of the network) and the arithmetic means of their x and y coordinates as an SBE’s *onset site* (**Figure 3A**, black circle). Onset sites were located throughout large parts of the network and clustered in some areas (**Figure 3B**). We identified distinct BIZs that repeatedly triggered SBEs during the recording period (NW_1_: *N* = 1887 SBEs in *t* = 165 min; 11.4 SBEs/min, 28 BIZs; NW_2_: *N* = 2141 SBEs in *t* = 192 min; 11.15 SBEs/min, 33 BIZs) by spatial clustering (centroid clustering with a cut-off at 1 mm) of onset sites (**Figure 3C**). The nine most prominent BIZs triggered the majority of SBEs (NW_1_: 83%, total 28 BIZs, **Figures 3D,E**; NW_2_: 74%, total 34 BIZs, Supplementary Figures S1D,E). To assess how compact BIZs were, we determined the probability of overlap of onset electrodes across individual SBEs of each BIZ, i.e., their probability for repeated participation in the onset of the SBEs triggered at a BIZ. Onset electrodes of SBEs triggered at a given BIZ showed a high degree of overlap (**Figure 3F**; note the logarithmic scaling).

The presence of distinct BIZs suggested that these network areas were characterized by some property supportive for SBE initiation. To gain more insight into possible mechanisms, we determined the ratio between mean local AFRs in BIZs and network-wide mean AFR (relative AFR) as a proxy for the relative local excitability (**Figure 3G**, upper panel). AFRs in BIZs were slightly higher in some but not all BIZs (NW_1_: 1.52 ± 0.07 Hz, *p* = 0.038; NW_2_: 4.27 ± 0.18 Hz, *p* = 0.03; mean ± SEM) but on average higher in the nine most prominent BIZs (NW_1_: 1.71 ± 0.02 Hz, *p* = 0.008; NW_2_: 4.32 ± 0.04 Hz, *p* = 0.14) compared to the global average (NW_1_: 1.33 ± 0.04 Hz; NW_2_: 3.83 ± 0.10 Hz). However, AFRs in BIZs were significantly lower than at the 25% most active electrodes (NW_1_: 2.89 ± 0.09 Hz, *P* = 8.23 × 10^-30^; **Figure 3G**, bottom panel; NW_2_: 7.94 ± 0.15 Hz, *P* = 1.3 × 10^-41^; Supplementary Figure S1G, bottom panel). The spatial map of relative AFR revealed hot and cold spots of activity in the network (NW_1_: **Figure 3H**; NW_2_: Supplementary Figure S1H). BIZs appeared to be mostly located on transitions between smaller hot spots surrounded by cold zones and were completely lacking in the largest contiguous hot zone.

We next asked whether BIZs were in a more active state when triggering SBEs. To assess this, we compared the burst strength (average number of spikes per BIZ electrode) when BIZs were actively triggering SBEs or passively recruited during SBEs (NW_1_: **Figure 3I**; NW_2_: Supplementary Figure S1I). Overall, SBE initiation was not paralleled by major increases in burst strength in the BIZ. In the first network, bursts were slightly stronger in the nine most prominent BIZs when these were triggering SBEs, however, there were no significant differences in the second network (NW_1_: ΔZ = 0.28, *p* = 1.2 × 10^-26^, **Figure 3I**; NW_2_: ΔZ = 0.02, *p* = 5.5 × 10^-1^, Supplementary Figure S1I; **Table 1**).

**Table 1:** Burst strength at BIZ electrodes when triggering synchronous bursting events (SBEs) (active) or when they were passively recruited into SBEs triggered elsewhere.

				BIZ index						
	1	2	3	4	5	6	7	8	9	pooled
**Network 1, PKC^N^, 21 DIV**					
passive	7.3 ± 2.3	12.8 ± 5.6	9.7 ± 3.0	5.3 ± 2.4	7.0 ± 2.9	6.2 ± 2.8	8.1 ± 2.8	10.6 ± 3.4	8.7 ± 4.5	
active	8.9 ± 3.6	15.6 ± 4.4	9.8 ± 2.1	5.5 ± 1.4	7.5 ± 2.3	6.4 ± 1.9	8.4 ± 1.9	10.5 ± 2.7	11.7 ± 3.5	
Δ	1.7	2.8	0.1	0.3	0.4	0.2	0.4	-0.1	3.0	
ΔZ	0.63	0.50	0.04	0.12	0.16	0.07	0.13	-0.01	0.66	0.28
p	1.1 × 10^-25^	1.4 × 10^-14^	5.9 × 10^-1^	1.3 × 10^-1^	5.0 × 10^-2^	3.8 × 10^-1^	2.2 × 10^-1^	9.0 × 10^-1^	8.0 × 10^-8^	1.2 × 10^-26^
n	327	271	219	177	172	165	97	70	68	1566
**Network 2, PKC^N^, 14 DIV**					
passive	12.6 ± 11.4	27.4 ± 20.2	35.9 ± 31.8	16.8 ± 12.5	19.0 ± 16.3	15.8 ± 13.5	26.9 ± 20.4	22.1 ± 19.1	20.7 ± 16.1	
active	15.1 ± 5.9	39.5 ± 31.9	18.2 ± 13.0	13.2 ± 12.4	18.8 ± 23.2	12.0 ± 9.4	19.0 ± 12.6	24.8 ± 13.4	17.6 ± 7.0	
Δ	2.6	12.1	-17.8	-3.6	-0.2	-3.8	-7.9	2.7	-3.1	
ΔZ	0.24	0.53	-0.57	-0.29	-0.01	-0.28	-0.39	0.14	-0.19	0.02
p	4.7 × 10^-6^	2.2 × 10^-19^	3.8 × 10^-15^	7.0 × 10^-4^	8.9 × 10^-1^	6.1 × 10^-3^	1.6 × 10^-4^	2.6 × 10^-1^	1.3 × 10^-1^	5.5 × 10^-1^
n	448	337	205	150	120	99	97	66	62	1584
**Network 3, PKC**^-^**, 24 DIV**					
passive	33.3 ± 26.1	33.3 ± 22.2	32.6 ± 22.3	23.2 ± 12.6	31.5 ± 22.9	15.9 ± 6.0	18.0 ± 9.1	11.0 ± 5.4	23.6 ± 15.2	
active	23.4 ± 2.7	27.9 ± 4.4	36.1 ± 22.9	29.6 ± 21.2	25.1 ± 2.3	22.9 ± 12.4	17.8 ± 8.3	15.8 ± 10.1	22.1 ± 6.3	
Δ	-10.0	-5.4	3.5	6.4	-6.4	7.0	-0.3	4.8	-1.6	
ΔZ	-0.46	-0.26	0.15	0.47	-0.29	1.05	-0.03	0.83	-0.10	-0.03
p	1.0 × 10^-6^	6.5 × 10^-2^	3.0 × 10^-1^	3.6 × 10^-3^	9.0 × 10^-2^	6.8 × 10^-7^	9.0 × 10^-1^	2.5 × 10^-4^	7.1 × 10^-1^	5.5 × 10^-1^
n	170	59	51	42	37	23	22	20	13	437
**Network 4, PKC**^-^**, 14 DIV**					
passive	19.1 ± 20.1	26.0 ± 24.5	22.1 ± 20.0	19.6 ± 16.9	21.1 ± 21.8	22.4 ± 19.7	21.8 ± 22.4	21.1 ± 18.8	16.5 ± 15.6	
active	21.8 ± 5.6	20.5 ± 26.9	20.6 ± 30.6	17.7 ± 20.9	23.7 ± 19.9	20.9 ± 25.3	21.6 ± 19.6	20.8 ± 7.9	24.3 ± 22.1	
Δ	2.7	-5.5	-1.5	-1.9	2.7	-1.5	-0.2	-0.4	7.8	
ΔZ	0.15	-0.22	-0.07	-0.11	0.12	-0.08	-0.01	-0.02	0.49	0.01
p	1.4 × 10^-1^	8.0 × 10^-2^	6.6 × 10^-1^	5.4 × 10^-1^	5.4 × 10^-1^	7.3 × 10^-1^	9.7 × 10^-1^	9.4 × 10^-1^	4.7 × 10^-2^	8.4 × 10^-1^
n	124	74	41	31	26	21	18	18	17	370


*Passive/Active: the mean ± SEM of the number of action potentials (AP) per electrode and SBEs; Δ, Difference in burst strength between active and passive condition; ΔZ, Relative difference between active and passive condition after z^_^scoring burst strength distributions for each BIZ; p, Significance level assessed with independent Student’s *t*-test after z-scoring (prior to pooling data). Individual and pooled data is shown for the nine most prominent BIZs in each network.*

Synchronous bursting events triggered by the same BIZ displayed similar propagation patterns resulting in correlated FSRO patterns (NW_1_: 0.68 ± 0.13, mean ± STD; **Figure 3J**; NW_2_: 0. 81 ± 0.13, Supplementary Figure S1J). To visualize the common patterns of SBEs triggered by a specific BIZ, we calculated the average FSRO patterns for the respective SBEs. On average, SBEs spread from the BIZs with a circular wave front with varying direction toward the network center (NW_1_: **Figure 3K**; NW_2_: Supplementary Figure S1K). FSRO correlation dropped with the distance between BIZs (NW_1_: **Figure 3L**; NW_2_: Supplementary Figure S1L) leading to negatively correlated recruitment patterns from BIZs at opposed sites of the network (distance 1 cm).

Their spatial extent suggests that BIZs are largely determined by local connectivity patterns supporting SBE initiation and their embedding into the overall network. The diminishing potential for large-scale remodeling after network maturation predicts that BIZs should remain stable over longer periods of time. To test this, we recorded the same network one week later (28 DIV) and determined the positions of BIZs during spontaneous SBE activity. The distribution of SBE onset sites indeed showed high resemblance between both recordings (NW_1_: **Figures 3B,M**) and several major BIZs were preserved with slight changes in their relative dominance in driving SBEs (NW_1_: **Figure 3N**). Similarly, we found a high correlation in the distribution of relative activity levels with hot and cold zones in the network one week after the previous recording (NW_1_: **Figure 3O**).

At 14 DIV, the PKC^N^ network (NW_2_) displayed superbursts (Wagenaar et al., 2006), i.e., phases with high frequent bursting (Supplementary Figure S1M) in which a single BIZ dominated the SBE initiation processes. This BIZ was localized within a relatively cold area of the network and also triggered regular SBEs outside of the superburst periods.

### Initiation and Propagation of Activity in Homogeneous PKC-Inhibited Networks

**FIGURE 4:**
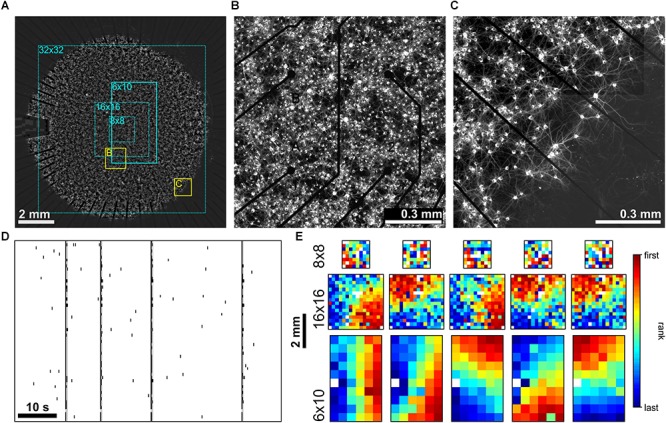
**(A)** PKC^-^ networks (∼150,000 neurons on ∼100 mm^2^) displayed a much more homogeneous arrangement of cell bodies and dendrites in central and boundary regions. **(B)** Zoom into the central region of the network recorded by the MEA (yellow rectangle in A). **(C)** Homogeneity in the arrangement of cell bodies and neurites extended to the boundary of the network (yellow rectangle in A). Dendrites extended beyond the cell body boundary and did not fasciculate as in PKC^N^ networks. **(D)** MEA Recordings of SBE activity at 21 DIV (network shown in A). **(E)** Exemplary SBE propagation patterns assessed by 8 × 8 (top row), 16 × 16 (middle row) and 6 × 10 (bottom row, network in A) MEAs in different PKC^-^ networks at 20–30 DIV. Color codes for the rank order of first spikes on an electrode in the course of SBEs. SBE origins were mostly located outside of the area recorded.

The preservation of BIZs over longer periods of time suggested that structural features, e.g., the connectivity patterns, were main determinants for the initiation of SBEs. This suggested that overall network architecture could have a crucial impact on shaping BIZs. To test this we manipulated network architecture by chronic inhibition of PKC as described previously (Okujeni et al., 2017). Chronic PKC inhibition (PKC^-^) impaired cell migration and activity-dependent neurite pruning during development, and led to a much more homogeneous distribution of cell bodies and neurites in mature networks. To gain insight into the influence of network structure on the initiation and propagation of SBEs, we recorded these networks with different MEA layouts and spatial scales (**Figure 4A**). In PKC^-^ networks, structural homogeneity was high in the center of the network compared to the clustered PKC^N^ networks (**Figure 4B**) and extended toward the boundary where dendrites extended beyond the sharp cell body border (**Figure 4C**). As reported earlier (Okujeni et al., 2017), these networks likewise generated SBEs (**Figure 4D**) that had their origin in a localized network area and then recruited large parts of the network. Recruitment patterns in PKC^-^ networks were much more homogenous than in PKC^N^ networks (**Figure 4E**). The highly isotropic wave fronts allowed extrapolating putative BIZs that were mostly located outside of the central MEA area.

**FIGURE 5:**
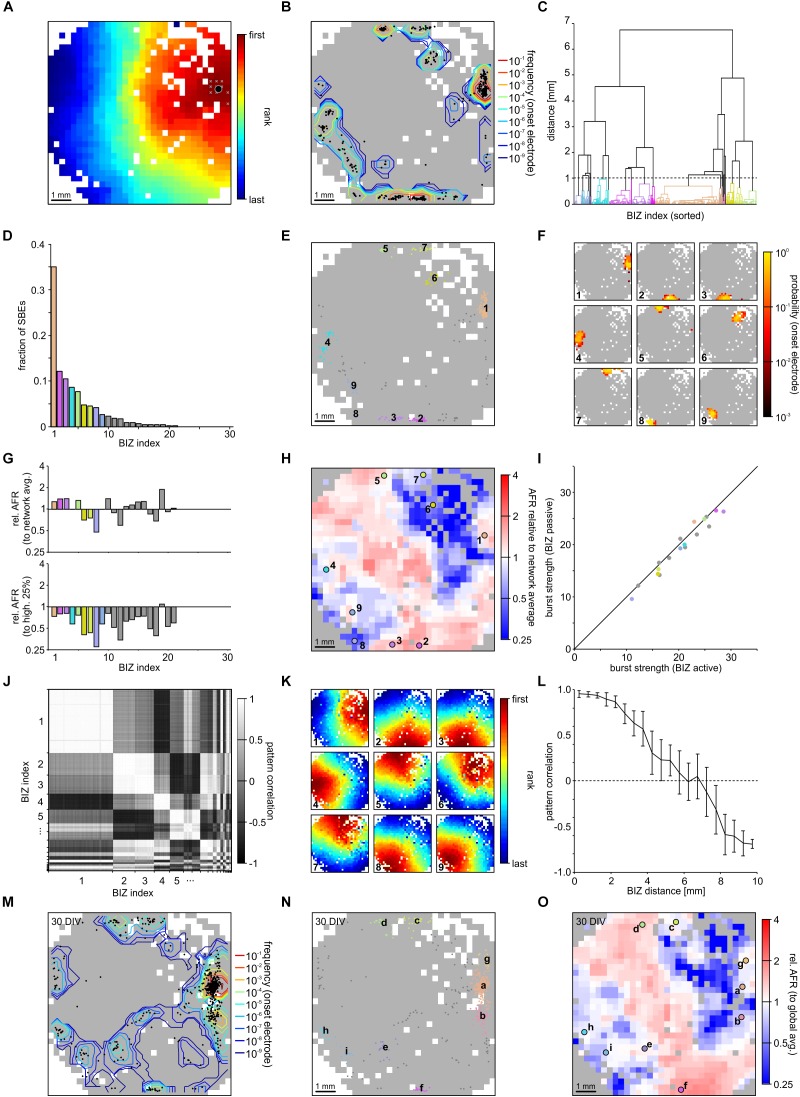
Synchronous bursting events dynamics in a PKC^-^ network at a 24 DIV (NW_3_). **(A)** SBEs were initiated in a much narrower region than in PKC^N^ networks and propagated across the network in a much more homogeneous fashion (3 × 3 median filter smoothing). White crosses mark the first ten recruited electrodes in a sample SBE and the black spot their means of the x and y coordinates defined as onset location. **(B)** Onset locations were located predominantly along the boundary and formed distinct BIZs (*N* = 486 SBE, *t* = 103 min; 4.7 SBE/min). Contour lines indicate the frequency with which individual electrodes were among onset electrodes (smoothed by 3 × 3 median filtering). **(C)** BIZs were identified by spatial centroid clustering of onset locations (cut-off at 1 mm distance between onset locations). **(D)** Burst initiation was dominated by even fewer BIZs than in PKC^N^ networks. The nine most frequent BIZ are color coded. **(E)** Mapping individual SBE onset locations shown in B to their respective BIZ (color code as in D) indicates that dominating BIZ lay close to but not at the network boundary. **(F)** BIZs reflected the centers of burst onset regions, which were mostly compact but not confined to extremely localized positions. The maps show the probability by which electrodes were among the first ten onset electrodes of bursts starting within the dominating BIZs. **(G)** As in PKC^N^ networks, average relative activity levels at BIZ electrodes (ratio of the mean AFR at BIZ electrodes and of all other electrodes with spike activity) were slightly above network average in the dominating BIZs and always lower than the 25% of highest AFRs. **(H)** Map of relative activity levels (ratio between the AFR at individual electrodes and network AFR during SBEs). BIZs appeared mostly located between hot and cold spots. Note that the large central region with high relative activity levels never initiated SBEs. **(I)** Median burst strength at BIZ electrodes when driving SBEs (active) or recruited during SBEs initiated by other BIZs (passive). Activity in the major BIZs was not significantly higher when they initiated SBEs. **(J)** Average propagation patterns elicited by the first nine BIZs revealed a homogeneous propagation of activity from different BIZ positions. **(K)** Similarity between propagation patterns was determined as the correlation of electrode recruitment ranks during SBEs. Sorting according to BIZ assignment revealed a very high correlation between propagation patterns originating at the same BIZs. **(L)** As in PKC^N^ networks, the correlation between propagation patterns dropped as function of distance between BIZs but yielded highly anti-correlated patterns for BIZ located at opposed sites of the network. **(M)** The distribution of SBE onset locations for the same network recorded one week later was highly similar (30 DIV, *N* = 627 SBE, *t* = 140 min; 4.5 SBE/min). **(N)** The overall distribution of BIZs was mostly preserved during development. Comparing letter sequence (reflecting decreasing frequency) to the numbers in H shows, however, that their influence had changed, focussing SBE initiation even more on one dominating BIZ. **(O)** Relative activity levels across the MEA displayed highly similar patterns at 24 and 30 DIV.

With small MEAs placed in the center of homogeneous PKC^-^ networks, BIZs appeared mostly located outside of the recording area and supposedly in boundary regions. To verify this we recorded homogeneous networks with 1k-MEAs. SBEs indeed typically originated in a quite narrow rim along the boundary of the network and propagated into the network with a homogeneous wave (Network 3 (NW_3_): **Figure 5A**; Network 4 (NW_4_): Supplementary Figure S2A). SBE onset sites were located mostly clustered in a few areas (NW_3_: *N* = 486 SBE, *t* = 103 min, 4.7 SBE/min, **Figure 5B**; NW_4_: *N* = 513 SBE, *t* = 49 min, 10.5 SBE/min, Supplementary Figure S2B), which yielded distinct BIZs after spatial clustering (centroid clustering with a cut-off of 1 mm; NW_3_: 21 BIZs, **Figure 5C**; NW_4_: 33 BIZs, Supplementary Figure S2C). SBE initiation, however, was dominated by a single BIZ (NW_4_: **Figure 5D**; NW_4_: Supplementary Figure S2D). The nine most prominent BIZs accounted for the majority of SBEs (NW_3_: 89.9%. NW_4_: 72.1%) and were arranged along or close to the boundary (NW_3_: **Figure 5E**; NW_4_: Supplementary Figure S2E). Onset electrodes of individual SBEs assigned to a particular BIZ had a high overlap probability (NW_3_: **Figure 5F**; NW_4_: Supplementary Figure S2F) within a compact core zone.

As in PKC^N^ networks, AFRs in BIZs were slightly higher in some but not all BIZs (NW_3_: 1.98 ± 0.08 Hz, *p* = 0.3; NW_4_: 3.46 ± 0.13 Hz, *p* = 0.23; mean ± SEM) and not significantly different in the nine most prominent BIZs (NW_3_: 1.95 ± 0.01 Hz, *p* = 0.67; NW_4_: 3.77 ± 0.02 Hz, *p* = 0.06) compared to the global average (NW_3_: 1.90 ± 0.04 Hz; **Figure 5G**, top panel; NW_4_: 3.27 ± 0.08 Hz; Supplementary Figure S2G, top panel). Likewise, AFRs in BIZs were significantly lower compared to the 25% of highest AFRs (NW_3_: 3.30 ± 0.05 Hz, *p* = 9.1 × 10^-43^; **Figure 5G**, bottom panel; NW_4_: 6.59 ± 0.11 Hz, *p* = 1.5 × 10^-54^; Supplementary Figure S2G, bottom panel). As in the PKC^N^ networks, the spatial AFR distribution revealed hot and cold zones of activity (NW_3_: **Figure 5H**; NW_4_: Supplementary Figure S2H) and again BIZ tended to be located at edges between hot and cold zones, and were completely lacking in the largest contiguous hot zone. In these networks, burst strengths were not systematically different for active and passive modes of BIZs (NW_3_: ΔZ = -0.03, *p* = 5.5 × 10^-1^, **Figure 5I**; NW_4_: ΔZ = 0.01, *p* = 8.4 × 10^-1^, Supplementary Figure S2I and **Table 1**).

Individual SBEs triggered from the same BIZs showed a very high correlation in FSRO (NW_3_: 0.95 ± 0.04, mean ± STD, **Figure 5J**; NW_4_: 0.85 ± 0.12, Supplementary Figure S2J) and those triggered from different BIZ only varied in the orientation of the circular wave front, suggesting highly homogeneous connectivity (NW_3_: **Figure 5K**; NW_4_: Supplementary Figure S2K). FSRO correlation dropped more conspicuously with the distance between BIZs than in PKC^N^ networks and BIZs at opposed sites of the network generated anti-correlated recruitment patterns (NW_3_: **Figure 5L**; NW_4_: Supplementary Figure S2L).

As in PKC^N^ networks, SBE pattern variability were thus determined by the position and predominance of BIZs. Compared to the PKC^N^ network, richness was strongly reduced since a single BIZ dominated the process.

The distribution of SBE onset sites was largely preserved when the homogenous network was recorded 6 days later (NW_3_:**Figure 5M**) and the BIZ prevailing at 24 DIV dominated SBE initiation even more at 30 DIV (**Figure 5N**). As in the PKC^N^ network, the spatial distribution of relative activity levels with hot and cold zones in the network was also preserved over time (NW_3_: **Figure 5O**).

### Common Aspects of SBE Initiation Across Networks

Across all networks, irrespective of their structure, AFRs tended to be slightly higher at BIZs compared to the network-wide average (+14.9 ± 10.7%; *p* = 0.043, Student’s *t*-test, *N* = 4 networks) but were significantly lower than at sites with the 25% highest AFRs (-42.5 ± 2.2%; *p* = 0.032, independent Student’s *t*-test, *N* = 4 networks). In addition, bursting was only slightly stronger on average when these were actively triggering SBEs than if passively recruited into SBEs (grand average across all networks: ΔZ = 0.12, *p* = 3.8 × 10^-12^), however, not consistently increased at all BIZs. Even some very prominent BIZs showed weaker bursts in their active role. The overall spatial distribution of AFRs across the network area typically revealed contiguous hot and cold zones with BIZs located at their transitions, suggesting that moderately rather than highly active network areas tended to trigger SBEs. The converse, however, was not the case, i.e., transition zones did not necessarily constitute BIZs.

## Discussion

In the current study, we analyzed how the sites of burst initiation relate to the mesoscale structure of neuronal networks by comparing networks with more irregular, respectively regular overall architecture.

### Large-Scale Network Sampling Provides New Insights Into SBE Dynamics

The mechanisms and network structures underlying SBE initiation and their propagation patterns in networks in culture have been discussed controversially. SBEs were described as a threshold-governed process within a scale-free connected network (Eytan and Marom, 2006). Consistent with this view, “leader” sites that fire early during spontaneous SBE were proposed to be part of subnetwork that is consistently activated during early stages of activity propagation (Eytan and Marom, 2006; Pan et al., 2009; Pasquale et al., 2017). As noted by Pasquale et al. (2017), these “leader” sites were mostly positioned at the edge of the small MEAs used, suggesting that BIZs might actually be located outside of the recording area and that the putative “leader” sites might only be stations along the SBE propagation pathway. Indeed, our findings using 1k-MEAs strongly support the latter interpretation and the hypothesis that SBEs are initiated locally in distinct BIZs.

Our recordings further show that the stereotypic wide-range propagation patterns mainly varied with the direction of the circular wave front and strongly depended on the position of the BIZ in the network. At a local scale, as observed with small MEAs, these patterns appeared noisy, in particular in PKC^N^ networks, implying a seeming richness of SBE patterns that cannot be extrapolated to the full network scale.

### SBE Initiation Occurs Within Distinct BIZs

Spontaneous network activity was proposed to emerge from background noise reflecting the stochastic nature of neurotransmission and ion channel state transitions, as well as the focussing and amplification of neuronal noise through convergent projections and recurrent connectivity motifs (Yarom and Hounsgaard, 2011; Orlandi et al., 2013; Lonardoni et al., 2017). In this view, SBE initiation occurs once the spatiotemporal summation of background noise reaches a critical threshold in one or a set of neurons (Maeda et al., 1995; Giugliano et al., 2004; Eytan and Marom, 2006).

Alternatively, pacemaker neurons (Gritsun et al., 2010) or highly active neurons (Shein et al., 2008) are considered to play a role in SBE initiation. Theoretical studies indeed suggested that bursting dynamics may emerge with low fractions of endogenously active neurons (Latham et al., 2000). Spiking activity in-between SBEs was very low in our networks. We did not find continuously firing neurons in BIZs. The relative increase of spiking activity in BIZ prior to SBE onset supports a process of local activity integration and amplification and argues against a dominating role of putative intrinsically active neurons for SBE initiation. Conceivably, we might miss such critical but rare neurons within BIZs given the sampling density of approximately 1:150 neurons per electrode. Yet, recordings with high density MEAs providing single neuron resolution likewise support SBE initiation by activity amplification within highly correlated local neuron ensembles (Lonardoni et al., 2017). The tendency of BIZs to be located at the boundary and at transitions between hot and cold zones nevertheless predicts that pacemaker neurons with specific biophysical properties would be influential only if they were suitably embedded into the network.

### BIZ Localization at Transitions Between Hot and Cold Zones

Burst initiation zones in our networks tended to be located along but not exactly at the boundary, which is consistent with calcium imaging studies with mini-cultures of up to 14,000 neurons growing on 20 mm^2^ (Orlandi et al., 2013). The propensity of the network boundary to shape BIZs suggests that this region promotes connectivity that fosters SBEs initiation. We speculate that this develops as a consequence of anisotropic connection opportunities and an increase of recurrent connectivity motifs at the boundary. These could focus and amplify activity that would otherwise remain below a threshold for burst initiation.

Comparing the BIZ locations in more homogeneous PKC^-^ and more inhomogeneous PKC^N^ networks showed that BIZs in PKC^N^ were more widely distributed across a network and also appeared in its center. Clustering, as in PKC^N^ networks, also introduces recurrent connectivity motifs and promotes spontaneous activity (Kaiser and Hilgetag, 2010; Klinshov et al., 2014), which would explain why BIZs were not exclusively located at the boundary in these networks.

Activity levels on BIZ electrodes, however, were close to the network average and well below the level of highly active recording sites. Furthermore, with respect to their own activity range, BIZs were not in a particularly high state of activity when driving SBEs. In fact, BIZs were conspicuously rare where average activity was particularly high (hot spots) or low (cold spots).

Combining these observations, we propose that the following connectivity scheme might lead to BIZs: Neurons positioned in boundary and transition regions are embedded anisotropically. On the one hand, they receive less input from the low-density boundary or regions between clusters, in consequence homeostatically upscale their synapses (Wilson et al., 2007; Okujeni et al., 2017) and form recurrent connections to maintain sufficient input levels necessary for neuronal survival (Van Ooyen et al., 1995). Local recurrent connectivity motifs could then amplify low-level activity as described by Lonardoni et al. (2017). Yet, this would be sufficient to initiate SBEs only if the output of this local network is well connected to recruit large parts of the network. Conversely, recurrent input from highly excitable regions to the BIZ must not be too strong to avoid lasting depression of excitability in the BIZ by SBEs (Weihberger et al., 2013; Kumar et al., 2016). A moderately connected position with locally recurrent connectivity would fulfill these prerequisites. Indeed, BIZ were mostly located at the transition between hot and cold spots of activity, supporting the notion that BIZs require a balance between independence and influence to trigger propagating SBEs. At high repetition rates, the propagation pathway fed by a BIZ would become less excitable, as shown for high stimulation rates by Eytan et al. (2003). Fatigue of this BIZ and its route of access to the network would then allow other BIZs to trigger SBEs.

### Richness of Activity Dynamics

Our results suggest that the richness of activity dynamics expressed in the variability of correlations between neuronal spiking crucially depends on the location and predominance of BIZs. Clustered networks had higher SBE rates that were driven by a large number of BIZs. Although some BIZs clearly dominated the process, some competition between BIZs was apparent and contributed to the richness of spontaneous activity patterns.

Richness was also promoted by the presence of distant BIZs producing anti-correlated propagation patterns. This was more pronounced in homogeneous networks with BIZs located very close to the boundary and more isotropic wave fronts. Nonetheless, this effectively did not increase richness of the SBE pattern distribution in these networks since a small number of BIZs strongly dominated the SBE generation process.

## Conclusion

Using 1k-MEAs we were able to record spontaneous activity across entire networks including their center and boundary. Our results indicate that SBEs in cultured networks are generally generated anew and do not emerge from continuing reverberations of activity at the boundary. BIZs remained stable over longer time scales and were located primarily at transitions between hot and cold zones. We suggest that the underlying structural framework and activity-dependent neurite growth shape recurrent connectivity in these areas. This promotes amplification of activity to a burst threshold determined by the state of excitability and associated refractoriness at that time. Our results suggest that transition zones in inhomogeneous networks could play an important role in governing network dynamics. The influence of the network boundary for connectivity and spontaneous activity generation may be particularly important to understand changes in network excitability following brain lesions.

## Author Contributions

SO performed research and analyzed the data. SO and UE designed research and wrote the manuscript.

## Conflict of Interest Statement

The authors declare that the research was conducted in the absence of any commercial or financial relationships that could be construed as a potential conflict of interest.

## Supplementary Material

FIGURE S1 Synchronous bursting events (SBE) dynamics in an immature PKC^N^ network at 14 DIV (NW_2_). At this stage, the network generated superbursts typical of immature networks, in addition to regular SBEs. **(A)** As in later stages, SBEs were mostly initiated in compact regions and propagated across the network from there (3 × 3 median filter smoothing). (White crosses: first ten recruited electrodes; black dot: means of the x and y coordinates defined as onset location). **(B)** Onset locations were distributed across large areas of the network but clustered in certain regions reflecting distinct BIZs (*N* = 2141 SBEs, *t* = 192 min; 11.1 SBE/min). Contour lines indicate the frequency with which individual electrodes were among onset electrodes (smoothed by 3 × 3 median filtering). **(C)** BIZs were identified by spatial centroid clustering of onset locations (cut-off at 1 mm distance between onset locations). **(D)** Fractions with which SBEs originated at a particular BIZ. For clarity only the nine most frequent BIZ are color coded. **(E)** Map of SBE onset locations in B assigned to their respective BIZ (color code as in D). Highly active BIZ lay close to but not at the network boundary. **(F)** Maps of the probability by which electrodes were among the first ten onset electrodes for individual BIZs. BIZs reflected the centers of burst onset regions. **(G)** Average relative activity levels at BIZ electrodes (ratio of the mean AFR at BIZ electrodes and of all other electrodes with spike activity). Activity levels in BIZs were similar to the network average but always lower than the 25% of highest AFRs. **(H)** Map of relative activity levels (ratio between the AFR at individual electrodes and network AFR during SBEs). BIZs were mostly located on transitions between hot and cold spots. Note that BIZ 1 clearly breaks this pattern. **(I)** Median burst strength at BIZ electrodes when driving SBEs (active) or recruited during SBEs initiated by other BIZs (passive). There was no noticeable connection between activity at BIZ electrodes in active, respectively passive mode. **(J)** Similarity between propagation patterns was determined as the correlation of FSRO. Sorting correlation coefficients according to BIZ assignment reveals a high correlation between propagation patterns originating in individual BIZs. **(K)** Average propagation patterns elicited by the nine most frequent BIZs. **(L)** The correlation between propagation patterns decreased with increasing distance between BIZs and yielded slightly anti-correlated patterns for BIZ located with larger separation. **(M)** Comparison of BIZs for regular SBEs and bursts that were part of a superbursts. Note that BIZ 1 strongly dominated SBE initiation during superbursts but not for regular SBEs.

FIGURE S2 Synchronous bursting events dynamics in an immature PKC^-^ network at 14 DIV (NW_4_). PKC^-^ networks rarely generated superbursts typical even at this stage. **(A)** As in mature networks, SBEs propagated across the network in a much more homogeneous fashion (3 × 3 median filter smoothing) than in PKC^N^ networks. (White crosses: first ten recruited electrodes; black dot: means of the x and y coordinates defined as onset location). **(B)** Onset locations were located predominantly along the boundary and formed distinct BIZs (*N* = 513 SBE, *t* = 49 min; 10.3 SBE/min). Contour lines indicate the frequency with which individual electrodes were among onset electrodes (smoothed by 3 × 3 median filtering). SBEs were initiated across a much wider region than at DIV 24. **(C)** BIZs were identified by spatial centroid clustering of onset locations (cut-off at 1 mm distance between onset locations). **(D)** Histogram showing the fraction with which SBEs originated at a particular BIZ with the nine most frequent BIZ color coded. **(E)** Map of SBE onset locations in B assigned to their respective BIZ (color code as in D). Highly active BIZ lay close to but not at the network boundary. **(F)** Maps of the probability by which electrodes were among the first ten onset electrodes for individual BIZs. BIZs reflected the centers of burst onset regions, which were more compact than in PKC^N^ networks. **(G)** As in PKC^N^ networks, average relative activity levels at BIZ electrodes (ratio of the mean AFR at BIZ electrodes and of all other electrodes with spike activity) were slightly above network average in the dominating BIZs and always lower than the 25% of highest AFRs. **(H)** Map of relative activity levels (ratio between the AFR at individual electrodes and network AFR during SBEs). BIZs were mostly located between hot and cold spots. Note that the large central region with high relative activity levels never initiated SBEs. **(I)** Median burst strength at BIZ electrodes when driving SBEs (active) or recruited during SBEs initiated by other BIZs (passive). Activity in the major BIZs was not significantly higher when they initiated SBEs. **(J)** Average propagation patterns elicited by the first nine BIZs revealed a homogeneous propagation of activity from different BIZ positions. **(K)** Similarity between propagation patterns was determined as the correlation of electrode recruitment ranks during SBEs. Sorting according to BIZ assignment revealed a very high correlation between propagation patterns originating at the same BIZs. **(L)** As in PKC^N^ networks, the correlation between propagation patterns dropped as function of distance between BIZs but yielded highly anti-correlated patterns for BIZ located at opposed sites of the network.
