# Supplementary figures and images for: Inhomogeneities in Network Structure and Excitability Govern Initiation and Propagation of Spontaneous Burst Activity
Source: Front Neurosci. 2019 May 31;13:543. doi: 10.3389/fnins.2019.00543 (PMC6554329; doi:10.3389/fnins.2019.00543)

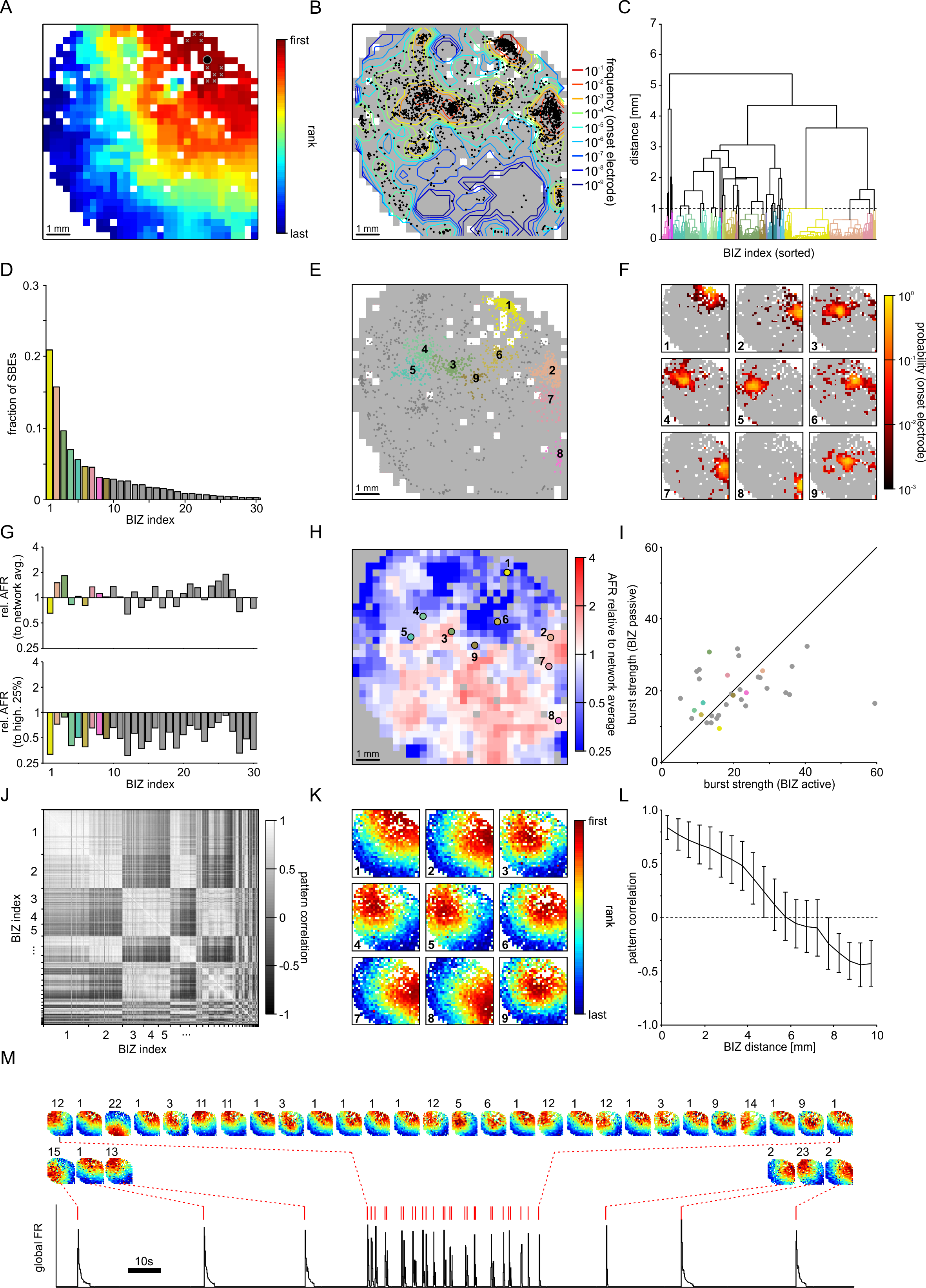

Supplement: FIGURE S1 — Synchronous bursting events (SBE) dynamics in an immature PKCN network at 14 DIV (NW2). At this stage, the network generated superbursts typical of immature networks, in addition to regular SBEs. (A) As in later stages, SBEs were mostly initiated in compact regions and propagated across the network from there (3 × 3 median filter smoothing). (White crosses: first ten recruited electrodes; black dot: means of the x and y coordinates defined as onset location). (B) Onset locations were distributed across large areas of the network but clustered in certain regions reflecting distinct BIZs (N = 2141 SBEs, t = 192 min; 11.1 SBE/min). Contour lines indicate the frequency with which individual electrodes were among onset electrodes (smoothed by 3 × 3 median filtering). (C) BIZs were identified by spatial centroid clustering of onset locations (cut-off at 1 mm distance between onset locations). (D) Fractions with which SBEs originated at a particular BIZ. For clarity only the nine most frequent BIZ are color coded. (E) Map of SBE onset locations in B assigned to their respective BIZ (color code as in D). Highly active BIZ lay close to but not at the network boundary. (F) Maps of the probability by which electrodes were among the first ten onset electrodes for individual BIZs. BIZs reflected the centers of burst onset regions. (G) Average relative activity levels at BIZ electrodes (ratio of the mean AFR at BIZ electrodes and of all other electrodes with spike activity). Activity levels in BIZs were similar to the network average but always lower than the 25% of highest AFRs. (H) Map of relative activity levels (ratio between the AFR at individual electrodes and network AFR during SBEs). BIZs were mostly located on transitions between hot and cold spots. Note that BIZ 1 clearly breaks this pattern. (I) Median burst strength at BIZ electrodes when driving SBEs (active) or recruited during SBEs initiated by other BIZs (passive). There was no noticeable connection between activi [file Image_1.TIF]

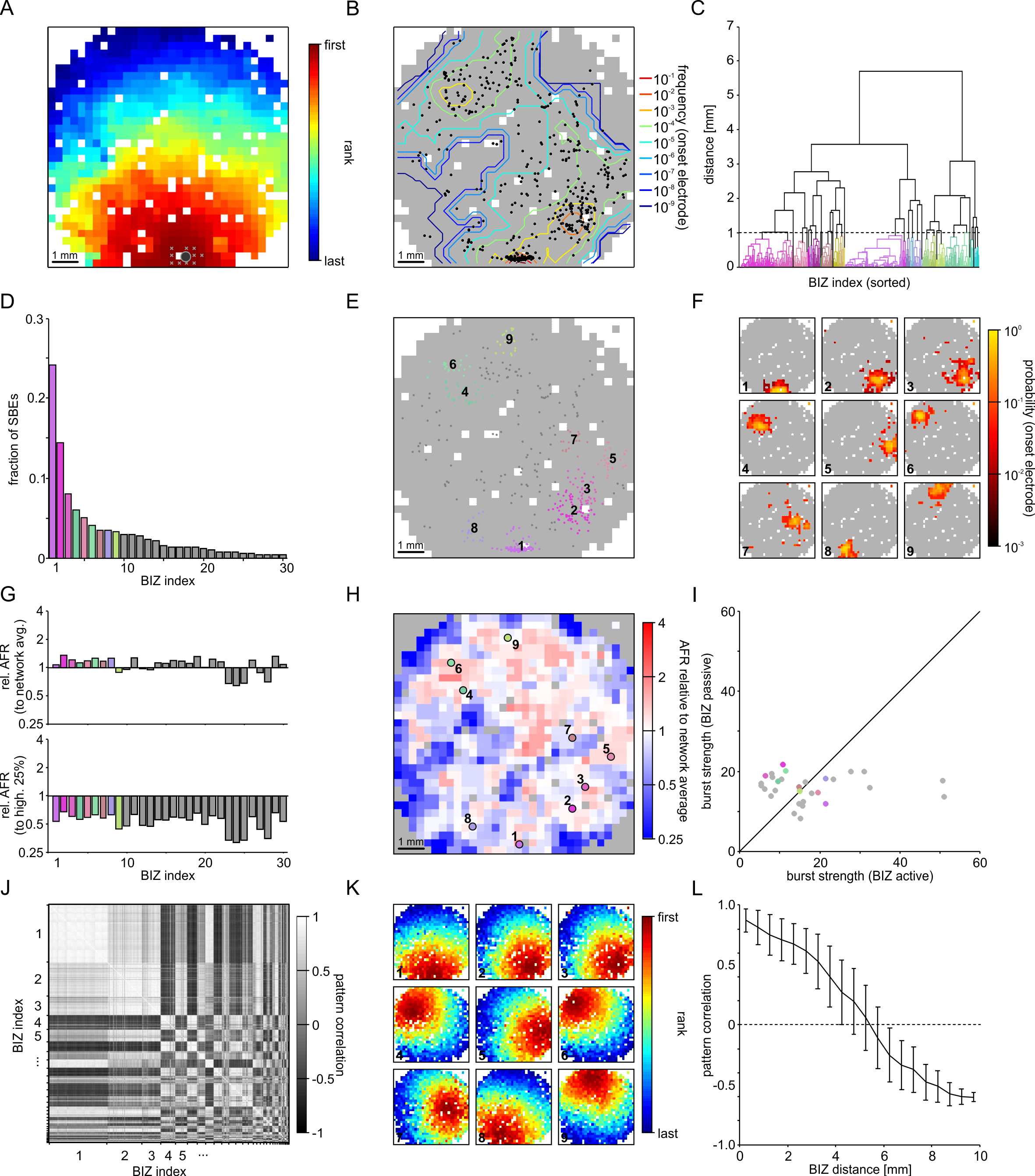

Supplement: FIGURE S2 — Synchronous bursting events dynamics in an immature PKC- network at 14 DIV (NW4). PKC- networks rarely generated superbursts typical even at this stage. (A) As in mature networks, SBEs propagated across the network in a much more homogeneous fashion (3 × 3 median filter smoothing) than in PKCN networks. (White crosses: first ten recruited electrodes; black dot: means of the x and y coordinates defined as onset location). (B) Onset locations were located predominantly along the boundary and formed distinct BIZs (N = 513 SBE, t = 49 min; 10.3 SBE/min). Contour lines indicate the frequency with which individual electrodes were among onset electrodes (smoothed by 3 × 3 median filtering). SBEs were initiated across a much wider region than at DIV 24. (C) BIZs were identified by spatial centroid clustering of onset locations (cut-off at 1 mm distance between onset locations). (D) Histogram showing the fraction with which SBEs originated at a particular BIZ with the nine most frequent BIZ color coded. (E) Map of SBE onset locations in B assigned to their respective BIZ (color code as in D). Highly active BIZ lay close to but not at the network boundary. (F) Maps of the probability by which electrodes were among the first ten onset electrodes for individual BIZs. BIZs reflected the centers of burst onset regions, which were more compact than in PKCN networks. (G) As in PKCN networks, average relative activity levels at BIZ electrodes (ratio of the mean AFR at BIZ electrodes and of all other electrodes with spike activity) were slightly above network average in the dominating BIZs and always lower than the 25% of highest AFRs. (H) Map of relative activity levels (ratio between the AFR at individual electrodes and network AFR during SBEs). BIZs were mostly located between hot and cold spots. Note that the large central region with high relative activity levels never initiated SBEs. (I) Median burst strength at BIZ electrodes when driving SBEs (active) or recruited during SBEs [file Image_2.TIF]
